# Perceptions and experiences of nurse managers of the implementation of the baby and mother friendly initiative in Namibia: a qualitative study

**DOI:** 10.1186/s13006-020-00336-2

**Published:** 2020-11-10

**Authors:** Justina N. Amadhila, Gisela H. Van Rensburg

**Affiliations:** Department of Health Studies, University of South Africa, Pretoria, South Africa

## Abstract

**Background:**

The baby and mother friendly initiative is a breastfeeding programme in Namibia aimed to protect, promote and support breastfeeding. The purpose of this study was to describe nurse managers’ perceptions and experiences of implementing the baby and mother friendly initiative in order to identify its successes and failures, as well as to develop guidelines for the strengthening of the programme.

**Methods:**

An evaluation research design to collect qualitative data through face-to-face interviews was conducted. A total of 33 interviews with nurse managers in charge of the baby and mother friendly hospitals, were conducted.

**Results:**

This study gave insight into the strength and weaknesses in the implementation of the programme implementation and make recommendations for improvement thereof. Four themes emerged from the study, namely: the extent of programme implementation, perceived benefits of the programme, challenges/hindrance to the implementation of the programme and recommendations for strengthening of the programme. The information was used to develop and validate guidelines that would help to strengthen the implementation of the programme.

**Conclusions:**

The study makes a contribution to the body of knowledge in nursing in that it provides guidelines for the strengthening of the baby and mother programme.

## Background

The Baby Friendly Hospital Initiative (BFHI) is a global initiative launched in 1991 [1]. The aim of the BFHI is to help health professionals protect, promote and support breastfeeding in health facilities. The promotion of breastfeeding is proven to reduce child mortality, improve nutritional outcomes and protect human capital [2]. The BFHI is implemented through the World Health Organization (WHO) and United Nations Children Fund (UNICEF)‘s Ten Steps to Successful Breastfeeding. These Steps are described as universally relevant as they apply to anywhere maternity services are offered in developed, developing, North, South, modern and traditional nations [1]. The steps are arranged in such a way that they depend on each other for successful breastfeeding. Table 1 presents a list of the BFHI Steps. In Namibia, the BFHI is renamed the Baby and Mother Friendly Initiative (BMFI). Namibia has adopted the Ten Steps to Successful Breastfeeding as the National Breastfeeding Policy [3]. The BMFI was launched in 1992. All 34 (100%) state and missionary state-subsidised hospitals were declared baby and mother friendly by 1997. This status was awarded after passing external assessments based on the Global BFHI Criteria. These hospitals are called Baby and Mother Friendly Hospitals. However, according to unpublished reports, the current status of BMFI implementation in these hospitals is not known as re-evaluation was never carried out. This lack of evaluation is despite the global and the Ministry of Health and Social Services (MoHSS)’ recommendations that baby-friendly hospitals should be re-evaluated at least every second year [4]. This paper reports the first phase, of the two phase study on the evaluation of the implementation of the BMFI programme in Namibia. In phase 1, interviews were conducted with nurse managers in charge of the Baby and Mother Friendly Hospitals to describe their perceptions and experiences of the implementation of the programme. The second phase comprised structured questions that attempted to ascertain how the BMFI hospitals in general, and in particularly the registered and enrolled nurses/midwives, were implementing the BMFI programme. Phase 2 will be reported separately.

**Table 1:** The Ten Steps to Successful Breastfeeding

(1) (a) Comply fully with the *International Code of marketing of Breast-milk Substitutes* and relevant World Health Assembly resolutions. (b) Have a written infant feeding policy that is routinely communicated to staff and parents. (c) Establish ongoing monitoring and data-management systems. (2) Ensure that staff have sufficient knowledge, competence and skills to support breastfeeding. (3) Discuss the importance and management of breastfeeding with pregnant women and their families. (4) Facilitate immediate and uninterrupted skin-to-skin contact and support mothers to initiate breastfeeding as soon as possible after birth. (5) Support mothers to initiate and maintain breastfeeding and manage common difficulties. (6) Do not provide breastfed newborns any food or fluids other than breast milk, unless medically indicated. (7) Enable mothers and their infants to remain together and to practise rooming-in 24 h a day. (8) Support mothers to recognize and respond to their infants’ cues for feeding. (9) Counsel mothers on the use and risks of feeding bottles, teats and pacifiers. (10) Coordinate discharge so that parents and their infants have timely access to ongoing support and care. (Source: WHO and UNICEF 2018)	

## Methods

### Study setting and participants

Thirty-four Baby and Mother Friendly Hospitals in 14 health regions were studied. A pilot interview was conducted in one of these hospitals, which was excluded in the main study. All participants were nurse managers in charge of the Baby and Mother Friendly Hospitals. Participants were recruited to interviews by telephone or email from the researcher. The researcher communicated the schedule of the field visits to the participants. Participants were requested to indicate possible suitable dates for appointments for interviews. On the day of interviews, participants gave individual verbal consent.

### In-depth interviews

A qualitative approach to data collection and analysis was followed. Face-to-face interviews were guided by an interview guide (see Table 2). The use of the interview guide ensures that the researcher can obtain all the information required and also gives participants the freedom to provide as many illustrations and explanations as they wish [5]. From the literature review, the concept of BMFI was operationalised to be measured into the WHO and UNICEF’s Ten Steps to Successful Breastfeeding. A concept can be measured by operationalising it from literature [6]. In preparation of the interviews the researcher had the following at hand: Copies of approval for the study from the MoHSS and University of South Arica, informed consent form, audio-tape recorder with extra batteries and a note book. The researcher explained the purpose of the study, voluntary participation and confidentiality of the information. With regard to anonymity, each participant was also informed that because of his or her position, a possibility existed that her or his identity could be recognised through the name of the hospital. The participants consented to the interviews as well as to the audio-tape recording thereof. In addition, participants were informed that an interview would take about 50 min. At the end of each interview, the researcher summarised the major points and asked the participants if they had any questions, as well as thanking them. An average interview lasted for 45 min. After each interview, the researcher listened to the audio-tape recording in order to familiarise herself with the data. Transcriptions were done in the evenings following each interview.

**Table 2:** Interview guide for phase 1

1. Your hospital has been declared baby and mother friendly some years back. Is Baby and Mother Friendly Initiative programme still being implemented in this hospital? 2. What is your experience with regard to the implementation of the Ten Steps to Successful Breastfeeding? 3. Which of the Ten Steps to Successful Breastfeeding do you regard as posing the most challenges to implementing the programme and why would you say so? 4. What would you regard as the strength of the BMFI programme? 5. What would you regard as the weaknesses in the implementation of the BMFI programme? 6. If the BMFI in this hospital needs improvement; what would you recommend?	

### Data analysis

Thematic analysis [7] was used to identify major themes and categories from transcripts. Coding was done manually. Codes were assigned manually and analysed in sub-categories and categories, which were grouped into themes. Themes were reviewed and refined by reading the entire set again in order to determine if there was a need for re-coding. In order to ensure rigour of data analysis, a second researcher coded the transcripts to see if the same categories emerged. Themes that emerged from the second researcher were included in the analysis.

## Results

### Demographic information of participants

The demographic profile of the participants, including gender, age group and years worked in current position of the participants is indicated in Table 3. There were 33 participants, of which 3 (9.1%) were males, while 30 (90.9%) were females. Seventeen (51.5%) nurse managers have worked for 5–10 years in their current positions, while 16 (48.5%) have worked for 11 to 15 years. Of a total number of 33 interviews that were conducted, one interview could not be concluded due to an emergency at the specific hospital. The nurse manager has confirmed the date and time of the interview before hand, however, she was called for theatre emergency in the middle of the interview. The interviewer could not complete the interview, as her return flight was booked for the same day.

**Table 3:** Demographic profile of the respondents (*n* = 33)

Characteristic	Value	Frequency
Gender	Male	3
	Female	30
Age group	30–39 years	0
	40–49 years	17
	50–59 years	16
	60 years and older	0
Years in current position	Less than 5 years	0
	5–10 years	17
	11–15 years	16
	More than 15 years	0

### Themes identified

**Fig. 1:**
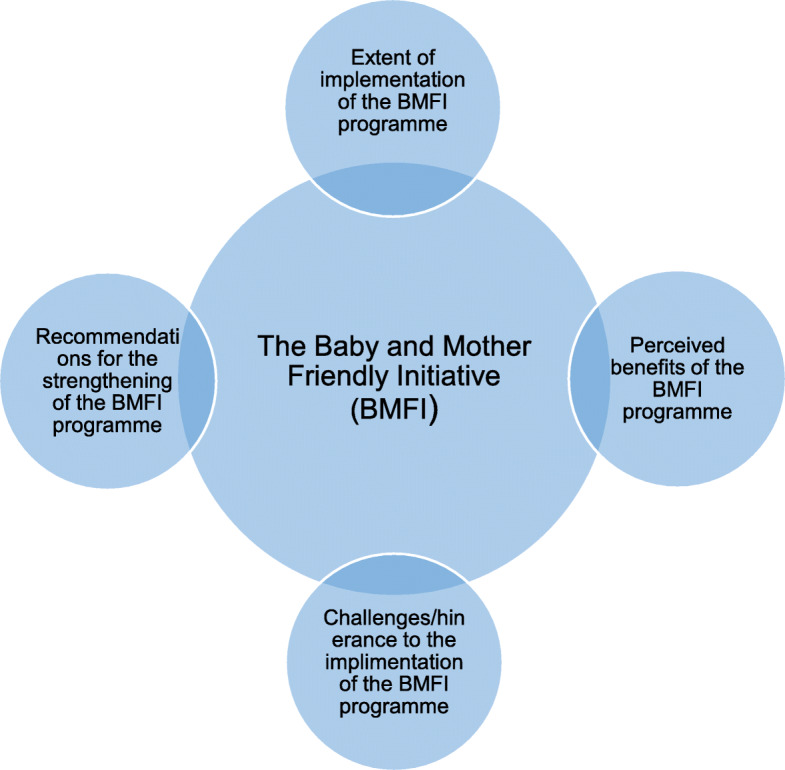
Themes identified from data

Four major themes with their categories and subcategories emerged from the data analysis. The themes were: 1. Extent of implementation of the BMFI programme; 2. Perceived benefits of the BMFI programme; 3. Challenges/hindrances to the implementation of the BMFI programme; and 4. Recommendations for the strengthening of the BMFI programme. These themes are illustrated in Fig. 1.

#### Theme 1: extent of implementation of the BMFI programme

*In theme 1, ‘extent of implementation of the BMFI programme’, the categories capacity and training emerged.*

Regarding the BMFI steps, the participants were quick to mention only a few of the ten steps to successful breastfeeding to justify their statements that the BMFI programme was implemented in their hospitals. Most frequently mentioned steps were: breastfeeding education for pregnant women (step 3), helping mothers to initiate breastfeeding (step 4), and rooming-in (step 7).

“[BMFI] is being implemented, − hmm − for me when it comes to the staff giving education to mothers, it is not a problem.” (Interview # 3).

“We are implementing it [BMFI] basically in maternity ward where we are initiating breastfeeding 30 minutes after delivery.” (Interview # 4).

“Yeah. .. because the babies are sleeping with their mothers, so, we still implement BMFI. Before that, every mother was having a baby-cot.” (Interview # 10).

When asked specifically about other steps, the participants acknowledged that some of the steps were not implemented. Partial implementation was evident.

“What step? No, no, that one was never implemented in this hospital.” (Interview # 27).

“Even when we started the initiative, there was no group [step 10] that was formed to support us. We have not done it.” (Interview # 10).

*In the category: capacity, the participants expressed the need for expertise and adequate funds for BMFI training.*

“I think we need a refresher course. We need the expert to come and train.” (Interview # 18).

“But I think the last workshop was in 2010. As from last year, because of other programmes pushing through [competing for resources], we did not have any formal workshop about breastfeeding.” (Interview # 20).

“We experience shortage of staff, it is also a challenge to send all staff to training.” (Interview # 19).

The participants also discussed capacity in terms of nurse shortages (the gap between the available number of nurses and the desired or the optimum number of nursing staff). A participant reported that the BMFI programme was no longer implemented in their hospital.

“Maybe [it] is because of shortages of staff. We are not really doing what we are supposed to do. No, no. no, to tell you the truth, BMFI is not implemented in this hospital.” (Interview # 16).

Most of the participants also confirmed that the BMFI programme was not well implemented.

“With staff shortage, you cannot really pay attention, especially to the primigravidas, to show them how to properly put babies on breast.” (Interview #7).

“. .. That is what is happening in this hospital. We are really, really understaffed, severely understaffed.” (Interview # 6).

#### Theme 2: perceived benefits of the BMFI programme

*In theme 2, perceived benefits of the BMFI programme, three categories emerged, namely healthy babies, reduced stigma of HIV/AIDS and improved breastfeeding practices.*

*The effect of breastfeeding on babies’ health and the protection against diseases were distinguished.*

“. .. Babies are very healthy; they are very nice, even if the mother is HIV positive they are followed up and. ..” (Interview # 33).

“These babies are not at risk of the many communicable diseases.” (Interview # 32).

*Regarding the category ‘reduced stigma of HIV/AIDS’*, the participants reported that the latest guidelines on infant feeding recommend breastfeeding for the general population, and also for babies born to HIV-infected women. It was reported that the implementation of these guidelines has contributed to the reduction of HIV-stigma-associated failure to breastfeed.

“The good thing is, stigma becomes less because everyone is breastfeeding.” (Interview # 2).

*Responses on ‘improved breastfeeding practices’ differed*.

Some participants believed that exclusive breastfeeding had increased owing to the BMFI programme. Others expressed concern about available reports showing low rates of exclusive breastfeeding in Namibia.

“I think breastfeeding practices, especially exclusive breastfeeding, has improved.” (Interview # 14).

“I have seen one report; they are saying that only 24 % of mothers are exclusively breastfeeding for six months. But all the hospitals are almost 100 %. They reach the target, but. .. , exclusive breastfeeding we are going down.” (Interview # 18).

#### Theme 3: challenges/hindrance to the implementation of the BMFI programme

The participants reported on various factors, which according to their experiences, influenced the implementation of the BMFI programme. *These factors emerged as categories ‘physical environment’, ‘perception regarding implementation of the BMFI programme’, ‘socio-economic factors’ and ‘teenage motherhood’.*

*The category physical environment was divided into subcategories; space and privacy*.

This category describes the physical capacity of the hospital and how it influences the BMFI programme activities.

*In the subcategory: space, participants expressed deep concern about availability of space in their hospitals.*

“Sometimes you find that there are a lot of deliveries, there is no space and you are forced to discharge some mothers, to make space.” (Interview # 22).

Regarding the subcategory: privacy, participants in small hospitals report that lack of separate wards for maternity patients made it difficult for them to implement the Ten Steps to Successful Breastfeeding.

“You know, the problem we are having here is the infrastructure itself. We have general ward, paediatric ward and delivery ward in-one. So, sometimes it is really difficult for our mothers and nurses to implement [the ten steps].” (Interview # 9).

*In the category: perception regarding implementation of the BMFI programme, the subcategories HIV and breastfeeding and effectiveness of the new guideline emerged.*

Participant shared their experiences that mothers fear the risk of transmitting HIV infection to their babies through breastfeeding.

“Most mothers are so worried, they want to breastfeed, but on the other hand they are afraid for the baby to get the virus.” (Interview # 31).

In addition, participants expressed concerns about the implications of the latest breastfeeding guidelines on health information education. They reported that when nurses give this the new information to mothers, they become uncomfortable because it conflicts with the previous messages. It was also reported that mothers also argued that the new information was not correct, based on their experiences with previous information.

“. .. Previously we were talking this, now we are talking about this, now you don’t really know.” (Interview # 32).

“They [women] tell you: I delivered my baby here four years ago but, and I was told, I must breastfeed for four months and. ..” (Interview #18).

Furthermore, participants reported that the huge shift in the recommendations on feeding babies of HIV-infected women, from replacement feeding in 2005 to full breastfeeding in 2011, has created uncertainty and doubt among health workers.

“The new guidelines, it seems, are doing well until now, although we don’t know the outcome.” (Interview # 3).

*The category ‘socio-economic factors’ was divided into the subcategories: education, affluence, race and employment*.

In this category, participants described factors outside hospital environments that challenge the implementation of the BMFI programme.

Participants reported that as a result of lack of education or poor education, mothers were not following instructions related to feeding their babies.

“Our people, you know, some mothers are not educated. You tell her this but tomorrow you find she is doing the thing you told her not to.” (Interview # 18).

”. .. but when they go home, they start using bottles, and. .. instead of cups.” (Interview # 27).

With regard to affluence, the participants experienced the fact that wealth influenced mothers’ feeding choices. They reported that some women regard breastfeeding as an option for those who could not afford to buy baby formula.

“People are thinking if they‘ve got money to provide milk – that affordability or the thought they can afford, is also contributing to infant formula use.” (Interview # 7).

“. .. These girls with money they don’t breastfeed.” (Interview # 33).

Regarding race, participants noted that white women prefer formula feeding above breastfeeding. They reported that even if white women know the benefits of breastfeeding, they choose formula feeding.

“Whites, they prefer bottle feeding, even if given information. .. they say they know. .. they are going on with bottle feeding.” (Interview # 5).

“Europeans, especially private patients, tell you: I am not going to breastfeed.” (Interview # 6).

Participants were critically of long working hours of breastfeeding mothers. They reported that factory workers and nurses work long hours per day, which makes it impossible for them to breastfeed their babies.

“I think it is more or less this thing; the mothers are working. At times, especially at our factories, the mothers are working from six-to-six o clock in the evening.” (Interview # 6).

“Because if they [nurses] work seven-to-seven, there is no time to go and breastfeed at home just one hour for lunch and come back.” (Interview # 32).

*Reasons such as going back to school, lack of understanding the benefits of breastfeeding and body image were reported as major role players in teenage mothers’ inability to breastfeed.*

Going back to school was the main reason reported that made it difficult for teenage mothers to breastfeed. For this reason, it was reported that teenage mothers did not even initiate breastfeeding (step 4 to successful breastfeeding).

“Teenage pregnancies is also contributing to these − not breastfeeding challenges, because these children want to go back to school and they don’t want to breastfeed.” (Interview # 7).

“. .. And they don’t see reasons why they should breastfeed for two days, [while in postnatal wards] as they will not continue.” (Interview # 1).

Regarding the subcategory: understanding, participants described that teenage mothers lack understanding of the importance of breastfeeding, hence they do not want to breastfeed.

“. .. Teenagers, they have to be encouraged. .. because they have to know the benefits. .. emphasise the benefit of the breastfeeding.” (Interview # 2).

“Teenage mothers, I don’t know if they don’t want or don’t understand, but sometimes they opt for bottle feeding.” (Interview # 9).

Participants noted that teenage mothers have a perception that breastfeeding has negative effects on their body shape. They reported that teenage mothers believed that breastfeeding would alter their body shape, especially their breasts.

“When it comes to teenagers – they tell you, they don’t want their breasts to sag” (Interview # 13).

#### Theme 4: recommendations for strengthening the BMFI programme

*In the fourth theme, namely strengthening the BMFI programme, the categories ‘development of healthcare professionals’, ‘community development’, ‘maternal support’ and ‘evidence based practice’ are discussed*.

Participants were asked whether they thought the BMFI programme in their respective hospitals needed improvement, and to share their opinions as to what could be done if that was the case. Training of health workers was regarded as a critical component of the BMFI programme. Participants suggested continuous training, including training for nurses and doctors at private facilities.

“Training for nurses is really important, especially those who are working in private [facilities] and the private doctors.” (Interview # 7).

“There should be ongoing training. People tend to forget, and new things come up every year. Yeah, for us to stay abreast with breastfeeding, we need regular training.” (Interview # 2).

“Because breastfeeding is important, we think when we are doing strategic planning we need to include breastfeeding training.” (Interview # 19).

The category ‘community development’ was divided into the subcategories: community education and community support groups.

With regard to community education, the participants emphasised the need for and importance of breastfeeding education of the community. They stressed that the focus of education should be on antenatal care services, private patients and awareness creation through the media.

“We have to strengthen health education at ante-natal care department. .. and encourage private patients to breastfeed, because private doctors are not in the action of preparation [preparing a mother for breastfeeding] properly.” (Interview # 7).

“Put emphasis on the benefits of breastfeeding. Give radio talks. .. through pamphlets to be distributed just to have information.” (Interview # 5).

Regarding the subcategory: community support groups, the participants expressed their concern about the absence of community-based breastfeeding support groups. They recommended formation of these groups.

“. .. But this one, with my manager we will sit down and talk about it, the support group.” (Interview # 4).

“Breastfeeding support group, it is something that we have to give attention on.” (Interview # 14).

*In the category: maternal support, the subcategory maternity leave emerged.* A participant was critical about the short period of 12 weeks paid maternity leave provided in the Namibia Labour Act of 2007. Those women who want more than 12 weeks have to take unpaid leave, which puts financial burden on them.

“I think maybe mothers can get, like in other countries, a full year of maternity leave. It will help.” (Interview # 6).

*In the category ‘evidence-based practice’*, a suggestion was made to conduct research on babies of HIV-infected women who are breastfed according to the new guidelines. It was reported that the research findings should be used to develop evidence-based guidelines for Namibia.

“My personal view, we need to conduct research so that we can. .. bring effective guidelines.” (Interview # 32).

## Discussion

In this study qualitative data was collected on the perceptions and experiences of nurse managers of the implementation of the BMFI programme. The information was used to develop and validate guidelines for the strengthening of the BMFI programme (see Table 4). These guidelines were validated by technical experts. Four themes emerged from the study, namely: the extent of implementation of the BMFI programme, perceived benefits of the BMFI programme, challenges/hindrance to the implementation of the BMFI programme and recommendations for strengthening of the BMFI programme.

**Table 4:** Summary of validated guidelines to strengthen the baby and mother friendly initiative

Theme	Category	Guideline
Extent of implementation of the BMFI programme	Capacity	• Improve working condition of staff
	Training	• Training
Perceived benefits of the BMFI Programme	Community development	• Community development.
Challenges/hindrance to the implementation of the BMFI programme	Physical environment	• Improving physical environment
	Perception regarding implementation of the BMFI programme	• Addressing perceptions regarding implementation of the BMFI
	Socio- economic factors	• Addressing socio-economic factors that affect mothers’ feeding choice
	Teenage motherhood	• Supporting teenage mothers to continue breastfeeding while schooling.
Recommendations for strengthening the BMFI programme	Development of healthcare professionals	• Development of healthcare professionals.
	Community development	• Community development
	Maternal support	• Maternal support

### Methodological limitations

The study was carried out in the Baby and Mother Friendly Hospitals in Namibia. The findings of the study can therefore not be generalised beyond these hospitals. This is also a limitation inherent to qualitative inquiry. In addition, health programmes can best be evaluated through interviews with those who are affected by them. In this study, the mothers are the ones affected by the BMFI policy. The quality of implementation could therefore not be determined as these findings are perspectives of a more homogenous group of people who might be influenced by sharing training experiences and biases.

### The extent of implementation of the BMFI programme

The findings of this study indicated partial implementation of the BMFI steps. The main reasons for partial implementation were inadequate capacity in terms of staffing and BMFI training. Staff shortage resulted in inadequate staff time to show mothers how to manage breastfeeding [8]. Healthcare staff should undergo 20 h training and three hours of supervised clinical training in breastfeeding management, in order for them to develop the knowledge and skills necessary to adequately support mothers on breastfeeding [9].

### Perceived benefits of the BMFI programme

Participants reported that because of the recent promotion of breastfeeding irrespective of the HIV status of mothers, the programme has contributed to the reduction of HIV stigma associated with non-breastfeeding, and to healthier babies and increased exclusive breastfeeding. Some participants noted that even though the hospitals were implementing the BMFI programme, available reports indicated low exclusive breastfeeding. Nevertheless, the Namibia Demographic and Health Surveys have shown that exclusive breastfeeding has increased from 6% in 2000 to 24% in 2006 and 49% in 2013 [10, 11]. The BMFI programme might have contributed to these improvements. Based on Demographic and Health Survey data, the impact of BFHI on breastfeeding was translated into healthcare savings associated with lower incidents of diarrheal and respiratory illnesses [12]. Breastfeeding interventions hold many benefits for the baby [13–15], and increase the rates of exclusive breastfeeding [16–18]. However, exclusive breastfeeding remained low in spite of breastfeeding interventions [19]. In order to increase the health benefits of breastfeeding, there is a need to educate the community on the importance of exclusive breastfeeding, as well as management of breastfeeding.

### Challenges/hindrance to the implementation of the BMFI programme

Participants reported that inadequate space, especially in smaller hospitals, made it difficult for nurses to implement most of the ten steps to successful breastfeeding, as well as to care for patients in privacy. Sometimes mothers were discharged early, before they were shown how to manage breastfeeding, in order to make space for newly admitted women. A mother who is not shown how to breastfeed may develop breast problem, which can lead to early stopping of breastfeeding. There is evidence that breast problems cause early termination of breastfeeding [20]. Inadequate space and lack of privacy made it difficult for health workers to counsel mothers on feeding [21].

It was reported that even though HIV-stigma associated with non-breastfeeding was reduced, some mothers feared the risk of transmitting HIV infection to their babies through breastfeeding. Fear of stigma and transmission of HIV infection to the baby caused mothers to breastfeed when in presence of others and give other milk of food when alone [22]. Mixed feeding increases the risk of transmission of HIV from mother to the baby [4]. These findings call for education of women, in order to address their perception on HIV and breastfeeding.

Participants reported that fear of HIV-infected women to transmit HIV infection to their babies, through breastfeeding, was based on earlier messages from health workers. They reported that regarding the latest guidelines, there is a huge change from emphasising replacement feeding in HIV cases to breastfeeding in spite of being HIV positive. This change of information is regarded as conflicting by both women and nurses, making the latter uncomfortable.

Participants described socio-economic factors outside hospital environment, such as education, affluence, race and employment, which influence breastfeeding practices. They reported that as a result of inadequate education, some women lack an understanding of instructions. Thus, they do not adhere to the instructions related to feeding their babies. Significant associations between higher education of women and increased exclusive breastfeeding have been identified elsewhere [22, 23]. It was reported that women who could afford to buy infant formula, especially white mothers and the affluent, perceived breastfeeding as an option for the poor. Contrary to the results of this study, literature shows the richer and richest wealth index is associated with exclusive breastfeeding practices [24]. Socio-economic challenges that influence mothers’ feeding decisions need to be identified and addressed.

Participants reported that teenage mothers do not breastfeed for several reasons, mainly the need to continue schooling, lack of understanding the importance of breastfeeding and a perception that breastfeeding spoils their breasts. Lack of knowledge of the benefits of breastfeeding and anticipation of difficulties associated with multiple roles of being a mother and student played a role in teenage mothers’ choice of feeding [24].

### Recommendations for strengthening the BMFI programme

Participants suggested that in order to strengthen the BMFI programme, there should be continuous professional development (CPD) in both public and private sector. Breastfeeding training equips health workers with knowledge and skills to educate women on how to manage breastfeeding [9]. This education would in turn lead to improved breastfeeding practices that would increase the rates of breastfeeding [25]. Continuous professional development is an ongoing commitment to lifelong learning to update, maintain, improve and develop knowledge required from all health professionals. Development of healthcare staff is a critical component of the BMFI programme. Training institutions should therefore design and offer accredited programmes in breastfeeding management.

Participants suggested that support for breastfeeding should be strengthened through community education. They reported that the focus should be on antenatal care services, the private sector and awareness creation through the media. They also acknowledged that establishment of community-based breastfeeding support groups would empower women to help one another. The importance of community involvement in supporting breastfeeding cannot be overemphasised; the majority (85%) of mothers received information on breastfeeding in their own homes and communities, and only 16% in a health facility [26].

Another suggestion was the extension of the current national maternity leave of 12 weeks to a full year. Mothers who took short maternity leave of 2 months had a higher risk of earlier stopping of breastfeeding than mothers who were still on maternity leave when the child was aged 10 months [27]. However, the suggestion of 1 year maternity leave has financial implications. Women regarded staying at home to breastfeed as a financial sacrifice [24]. The best way to balance breastfeeding and financial needs is therefore through the provision of support for breastfeeding in workplaces.

A suggestion was made to conduct research on babies of HIV-infected women who breastfed according to the new guidelines. It was reported that the research findings should be used to develop evidence-based guidelines for Namibia. This call for evidence might be an indication that participants were not aware that the new guidelines are based on the updated WHO recommendations on HIV and infant feeding [28] and research must have been conducted before these recommendations were issued. However, it is encouraged to see managers demanding evidence-based practice.

## Conclusions

This research paper is the only study that has examined the perceptions and experiences of nurse managers about the implementation of the BMFI programme in Namibia since 1997. The study gave insight into the strength and shortcomings in the implementation of the BMFI programme. The study makes a contribution to the body of knowledge in nursing in that it provides guidelines for the strengthening of the BMFI programme.

## Data Availability

The datasets used and/or analysed during the current study are available from corresponding author on reasonable request.
